# Perspective: Are We Ready to Measure Child Nutritional Status with Lasers?

**DOI:** 10.1093/advances/nmy053

**Published:** 2019-02-05

**Authors:** Joel Conkle, Reynaldo Martorell

**Affiliations:** 1 Nutrition and Health Sciences Program, Laney Graduate School, Emory University, Atlanta, GA; 2 Hubert Department of Global Health, Rollins School of Public Health, Emory University, Atlanta, GA

**Keywords:** anthropometry, body composition, child growth, development, nutritional assessment, nutritional surveillance

## Abstract

The continued use of basic, manual anthropometric tools (e.g., boards and tapes) leaves anthropometry susceptible to human error. A potential solution, 3-dimensional (3D) imaging systems for anthropometry, has been around since the 1950s. In the 1980s, 3D imaging technology advanced from photographs to the use of lasers for body digitization; and by the 2000s, the falling price of 3D scanners made commercial application feasible. The garment sector quickly adopted imaging technology for surveys because of the need for numerous measurements and large sample sizes. In the health sector, 3D imaging for anthropometry was not widely adopted; its use was limited to research and specialized purposes. The different cost and logistical requirements for measurement in the garment and health sectors help to explain why the technology was adopted in one sector and not the other. Despite reductions, the price of 3D imaging systems remained a barrier to the use of 3D imaging for regular nutritional assessment in the health sector. Additional barriers in the health sector were that imaging systems required dedicated space and were not designed for capturing measurements in young children. In recent years, the development of light-coding technology may have removed these barriers, and a handheld imaging system was developed specifically for young children. There are not yet recommendations to replace manual equipment with 3D imaging for nutritional assessment, and there is a need for more research on low-cost, handheld imaging systems—particularly research that evaluates the ability of 3D imaging to improve the quality of anthropometric data and indicators.

## Introduction

Anthropometry, or the measurement of the human body, is an ancient practice. Texts from Ayurvedic and traditional Chinese Medicine show that human beings have attributed meaning to variation in human surface morphology for thousands of years (1, 2). Anthropometric methods were standardized in science in the 18th and 19th centuries, and although interpretation of anthropometry changed dramatically over the centuries—with measurements applied to health and well-being, productivity, fighting ability, fortune-telling, and eugenics—methods and tools for anthropometry have changed little since the 1800s (2, 3). Today's anthropometric tools are rudimentary. We rely on wooden boards, tapes, and calipers, which are some of the same basic tools found in bc China (2).

Three-dimensional (3D) imaging is the norm for anthropometry used in garment design and ergonomics, but in the health sector the use of 3D imaging is limited to research and specialized purposes. We set out to determine why 3D imaging for anthropometry was used regularly in one sector and not the other. Along the way, we identified key barriers to the use of 3D imaging for regular nutritional assessment in the health sector and found that recent technological developments could be removing these barriers, making it more feasible to use 3D imaging for regular nutritional assessment, including for growth monitoring of young children.

## Why Is the Use of Manual Tools for Anthropometry a Problem?

New technology, such as DXA (4), improved the measurement of body composition but did little to improve anthropometry. The lack of advancement in anthropometric methods is problematic. First, current equipment, especially length/height boards, places a burden on anthropometrists and young children. In some settings, anthropometrists must carry bulky and heavy boards, and length measurements push a large percentage of young children into a crying fit. Second, for routine nutritional assessment, we still rely on a few basic measurements because many body measurements with known or potential diagnostic value are too difficult to measure with manual anthropometry and because the cost and complexity of laboratory techniques make laboratory measurement unsuitable for routine assessment. The third and most important reason that the lack of advancement is problematic is that current tools are susceptible to human error (5), and when manual tools such as measuring tapes and length boards are used outside of a research setting—when stringent training and quality control are not in place—the result is often poor-quality measurement, especially in young children.

High-quality manual anthropometry is labor intensive and is only possible with well-trained, diligent anthropometrists. It is no surprise that poor-quality anthropometry was documented extensively in health facilities and surveys in both developed and developing countries, with circumferences and lengths showing the worst reliability and accuracy (6–14). Measurement error is particularly common in children aged <3 y because many children in this age group will not stay still for measurement and may actively resist measurement, especially when asked to lie down on a length board. The result of human error is that anthropometric data quality varies among countries and among surveys in the same country, making it difficult to meaningfully compare countries, analyze trends over time, or target public health interventions. At the individual level, poor-quality anthropometry limits our ability to monitor growth and leads to misclassification of nutritional status during screening. The usefulness of anthropometry is undermined both by indicators of limited predictive power, as is the case for BMI, and by poor measurement quality, with the latter leading to calls from the global nutrition community for new technology to improve the quality of child anthropometry (11, 15).

## Our Introduction to 3D Imaging for Anthropometry

Our research team at Emory University recently responded to calls for new measurement technology (11, 15) by collaborating with the private sector on a validation study of a low-price, handheld 3D imaging system designed to measure child stature, arm circumference, and head circumference. The company that developed the software for the imaging system used in our validation study, Body Surface Translations, Inc., had extensive experience in measuring mobile subjects with 3D imaging, having spent 7 y developing and testing 3D scanners to estimate the weight of livestock on the basis of surface morphology. The company's experience in measuring moving subjects caught the eye of global nutrition experts because of the potential application to hard-to-measure children. The Bill and Melinda Gates Foundation brought together the company, which had no experience working with humans, with our research team—a team with experience in anthropometry, including experience in developing the 2006 WHO Child Growth Standards (16), but with no experience in 3D imaging—into a partnership to develop and test a 3D imaging system for child anthropometry (17–19).

## Historical Perspective: From Photographs to Lasers

As early as 1952, methods were developed to use a pair of facial photographs to create a rough, 3D representation of a human face (20). The use of 2-dimensional photographs for 3D reconstruction was extremely labor intensive, but processing was eventually automated (21). One of the earliest uses of automated processing of 3D photogrammetry for anthropometry was to assess the nutritional status of American astronauts; the researcher used a Cray supercomputer and software from the US Air Force that was designed for aerial mapping (M Golden, University of Aberdeen, personal communication, 2017) (22).

In the 1980s, 3D imaging for anthropometry took a technological leap, advancing from photographs to body digitization with lasers via “range imaging,” which is a blanket term covering various methods that project light onto the person being measured and use triangulation to construct a 3D surface map (21, 23). Range imaging was applied to anthropometry in the United States in 1986 (24); and around the same time in the United Kingdom another range imaging system was developed through a collaboration between a university and the garment manufacturing industry (25); industry would eventually bring 3D imaging to practical anthropometry applications.

## 3D Imaging in Sizing Surveys

In both the United States and the United Kingdom the major driver for continued development of 3D imaging systems for anthropometry was that sizing surveys, needed for garment design and ergonomics, were costly (24, 25). From car seats to khakis, products need to correspond to human dimensions, and sizing surveys have been used for decades to capture those dimensions. The garment industry wanted sizing surveys with large samples (4500–6500) and ∼40 measurements per subject; with these requirements, manual methods took too much time and money (25). By the late 1990s, a large-scale sizing survey, the Civilian American and European Surface Anthropometry Resource, employed the use of 3D imaging for anthropometry (24). The Civilian American and European Surface Anthropometry Resource survey collected data in the United States, The Netherlands, and Italy and was supported by >20 industrial partners (24).

By the 2000s, the price of 3D range imaging systems dropped dramatically, from hundreds of thousands of dollars to <$10,000, and 3D imaging became common in national sizing surveys around the globe (23, 26–28). In 2001, the SizeUK sizing survey used the TC2 scanner (TC^2^), and multiple countries then used the same or similar technology and the same naming convention for their own survey, giving us SizeUSA, SizeJapan, SizeKorea, SizeThailand, and others (26, 27, 29). In the first decade of the 21st century, 3D imaging became the standard for anthropometry in the garment sector, but the same cannot be said for the health sector.

## 3D Imaging in the Health Sector

3D imaging for anthropometry is not new to the health sector. In the past, medical researchers used photograph- or scan-derived 3D anthropometry for a variety of purposes, including to diagnose scoliosis, underdevelopment of the optic nerve, and melanoma; to assess treatment of skin ulcers; and to predict obstructive sleep apnea (30). In routine clinical practice, 3D imaging is used for orthotics and orthodontics (31, 32), including for young children. However, when looking at the experience of 3D imaging in the health sector it is important to distinguish between different types of commercial range imaging systems: custom systems; the type used in sizing surveys, which currently costs close to $10,000; and a newer type used in the gaming industry that costs <$1000.

Starscanner (Vorum) is a custom-built 3D scanner for newborn cranial remolding orthoses (32) that is now in hundreds of medical facilities, but it is large, expensive, and was not designed with regular nutritional assessment in mind. Custom 3D scanners are not practical for routine measurement.

Researchers in the health sector tested commercial range imaging scanners (type used in sizing surveys) for measurements relevant to the assessment of nutritional status, such as height (33), circumference (34–36), body surface and volume (26, 37, 38), and body shape (26, 39, 40). Some of the studies considered the use of 3D imaging in nutritional epidemiology. Jaeschke et al. (35) found that scan-derived measurements correlated as well as manual measurements with biochemical markers of metabolic syndrome, and Lin et al. (40) developed a new index from scan-derived waist, breast, and hip area—the Health Index—and found good correlation between the new index and biochemical markers of metabolic disorders. Over the past decade, researchers started to use the actual data from sizing surveys supported by industry for health research. In 2007, Wells et al. (26) examined associations between body shape and BMI using SizeUK data, and researchers in Thailand used SizeThailand data to study diabetes and obesity (29). Despite extensive health sector research on the use of “sizing survey type” 3D scanners for anthropometry, the work never translated into the use of 3D imaging for routine nutritional assessment.

**FIGURE 1:**
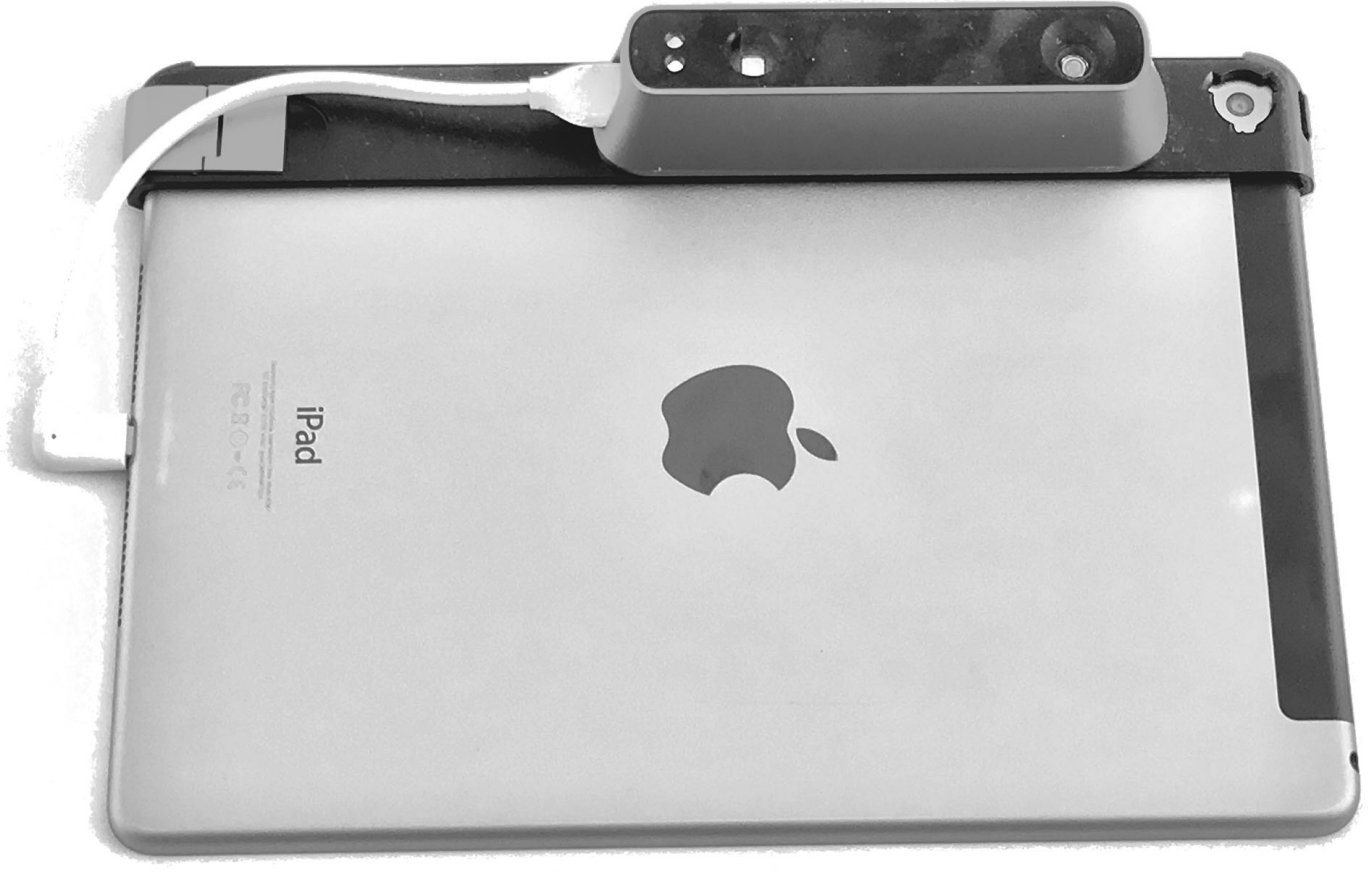
3D imaging system with tablet. Hardware setup is from Body Surface Translations, Inc., for the BINA. A structure sensor 3D scanner is connected to the tablet. BINA, Body Imaging for Nutritional Assessment Study; 3D, 3-dimensional.

**FIGURE 2:**
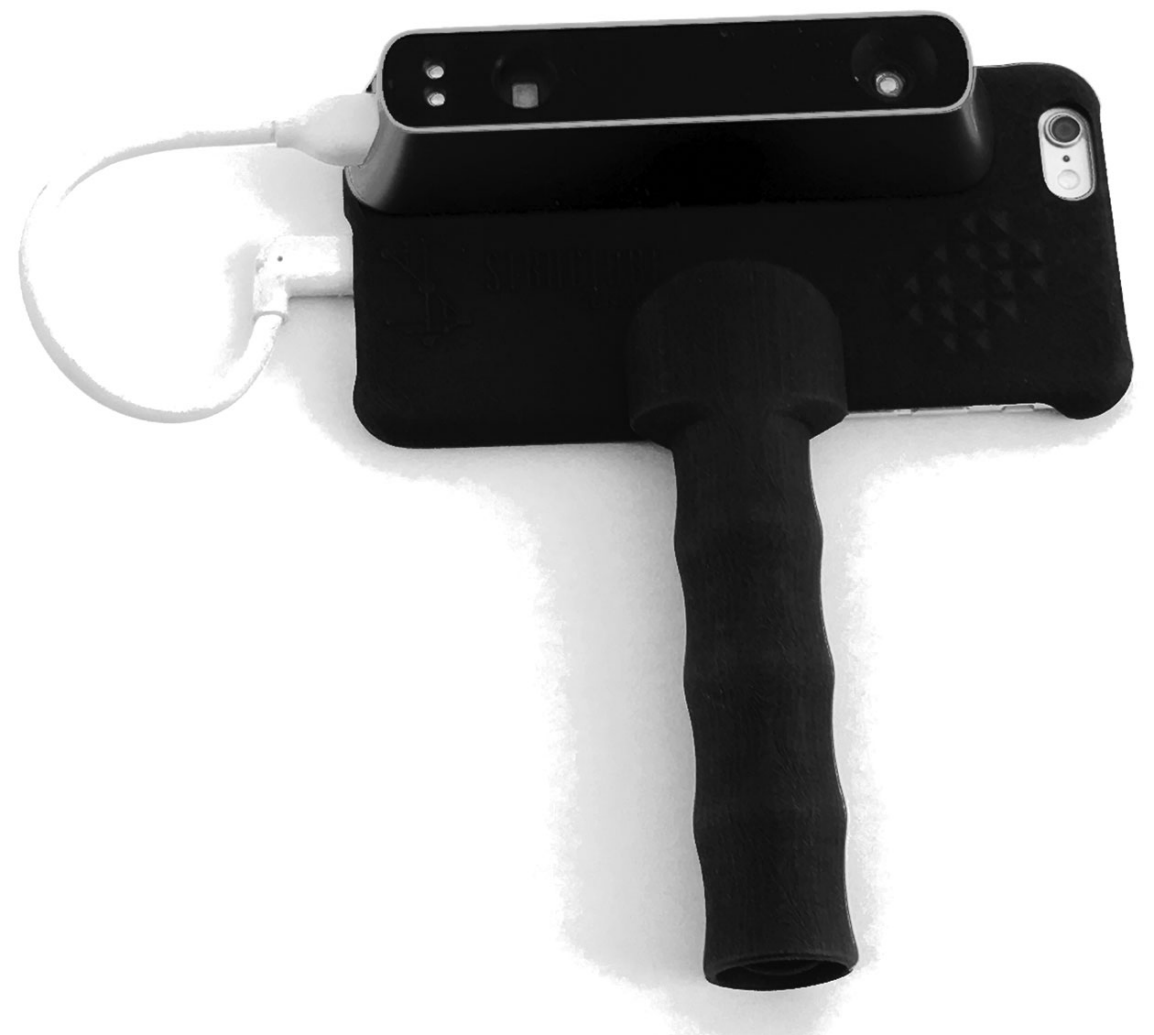
3D imaging system with phone. Prototype hardware setup is from Body Surface Translations, Inc. A structure sensor 3D scanner is connected to a phone. 3D, 3-dimensional.

In 2010, PrimeSense (acquired by Apple in 2013) licensed its “light-coding” technology for use in Microsoft Kinect. Light-coding is range imaging that requires a single device: an infrared projector and sensor are contained in the same device and stereo triangulation is achieved by comparing the sensor image to an image of the projector's pattern that is hardwired into a microchip. Light-coding reduced the price (<$1000) and size of 3D scanners and led to the use of 3D imaging in the gaming industry. A few studies were carried out to evaluate the use of Kinect for anthropometry. One study measured stationary cylinders as a proxy for human circumferences (41), another study compared Kinect to a more expensive range imaging system for various measurements (42), and a third study made estimates of body volume (42, 43). In our own validation study, we used lower-cost technology similar to the Kinect, but there were important differences between our study and previous studies. Our research used a single, handheld scanner that could be moved around (**Figures 1** and **2**), and our imaging system was designed specifically to measure young children, accounting for child movement by taking multiple short-duration scans and stitching them together. Imaging systems used in previous research with “Kinect devices” or “sizing survey devices” used multiple scanners in fixed positions and the systems were not designed for young children. A full description of the scanning protocol in our study is available in the study manual on Open Science Framework (44).

The results of our study showed that a lower-cost, single, handheld scanner produced measurements in children that were as reliable as gold-standard manual measurement (18). For accuracy, we found systematic differences of 2–6 mm when compared with manual measurements, but we concluded that a simple recalibration of the imaging software would likely fix the accuracy problem, and that it would soon be possible to accurately and reliably measure children with a single, handheld scanner using current technology.

## Barriers to the Use of 3D Imaging for Regular Nutritional Assessment

With 3D imaging the norm for anthropometry used by industry and a considerable amount of research on 3D imaging for nutritional assessment, the question arises: Why are 3D scanners not used for routine health checks? In the 1990s, in a review of digital photogrammetry, Mitchell (45) concluded that the requirements for the use of 3D photogrammetry in the health sector represent a “surprisingly low level of cost” and there is no need for a “workstation,” pointing out that surface morphology is not crucial to assessing a patient's health because of the availability of internal examination.

Decades later, the experience of the Body Benchmark Study (46) showed that the same barriers applied to the use of range imaging systems in the health sector. In the early 2000s, Select Research, a company that carried out sizing surveys for the garment industry, researched applications for 3D imaging in the health sector (46). In 2007, Select Research launched the Body Benchmark Study, a study that set out to replace anthropometric proxies of body composition, specifically BMI, with a new indicator derived from 3D scans, the Body Volume Index (46). The study results were made public in 2010, and the overall conclusion was that Body Volume Index offered advantages over traditional measures (46). In 2010, the UK National Health Services (NHS) reviewed the research and rejected a proposal to install 3D scanners across the NHS; the company reported that they were advised by the NHS to develop a low-cost, mobile solution (46).

Mitchell's review and the experience of the Body Benchmark Study showed that the technology used in sizing surveys did not adequately fulfill important criteria for adoption in the health sector—namely, low cost and no requirement for dedicated space. Range imaging systems that are commonly used in sizing surveys, like the 3D photogrammetry systems before them, require multiple cameras in a fixed position for stereo triangulation; the technology did not remove the need for a workstation and, although the technology was cost-effective for sizing surveys, it was still too expensive for regular nutritional assessment. An additional limitation of the type of range imaging systems used in sizing surveys was that the long scanning period (∼10 s) made it difficult to measure children, especially children aged <3 y, who move constantly and resist measurement.

The recent development of lower-cost, portable scanners may have already removed the “workstation” barrier. In our validation study with the newer scanners we measured children in health facilities by carrying a 3D scanner from room to room; there was no need for dedicated space because we did not set up multiple cameras in fixed positions. Our validation study also showed that it was possible to measure children reliably with imaging software designed specifically to handle movement. However, at the time of our validation study, the price of the 3D imaging system was $878 (tablet and scanner) compared with $122 for a length/height board (47). The price of 3D imaging systems was substantially higher than manual equipment, but the price of 3D scanners continues to decrease; 3D scanners are now being included in mobile phones for facial recognition, and there may be less of a difference now if we consider the entire cost of taking measurements. There is a need for a comprehensive costing study, and that research should consider all factors affecting cost, including training and staff needs, measurement time, and the potential value of 3D data to industries that currently carry out sizing surveys.

It appears that the identified barriers to the use of 3D imaging for regular nutritional assessment in the health sector were already addressed or will be addressed in the near future by technology development, but it is important to note that there is a lack of experience implementing 3D imaging for anthropometry in clinics, hospitals, and health surveys; and there may be additional barriers that were not yet identified. In our validation study, we identified characteristics of the imaging system that could potentially be additional barriers to adoption in the health sector: anthropometrists were not comfortable taking scans of uncooperative children and scanning required that subjects undress to their undergarments. We believe these additional barriers can be overcome: privacy concerns related to undressing can be resolved in most health sector contexts, scanning protocol could be adjusted to allow for some clothing depending on the types of measurements captured, and further software development can provide anthropometrists with more confidence. However, we will not know all of the barriers to adoption in the health sector until 3D imaging is put to use in everyday practice. Experience implementing 3D imaging for anthropometry is needed to evaluate the potential for widespread use of the technology.

## The Role of 3D Imaging in Addressing Limitations of Manual Measurement

### Reducing burden

When compared with manual equipment, a handheld, lightweight 3D imaging system obviously places less transport burden on anthropometrists, especially those working on surveys in remote areas. Anthropometrists in our study appreciated that the AutoAnthro system was ultraportable and well accepted by children (19), indicating superiority over manual equipment with respect to transport and invasiveness.

### Improving data quality

We did not identify any research that evaluated the ability of 3D imaging to improve anthropometric data quality. In our own validation study, some of the results suggested that 3D imaging may not be as susceptible to human error as manual equipment, but we were not able to draw any conclusions on improving data quality because we did not directly test quality improvement (18). There is a need to test the ability of 3D imaging to improve anthropometric data quality, and future research should take into account that improving body measurements may not remove all measurement error in a developing-country context because many anthropometric indicators include age and in many countries misreported age is a common source of error (14).

While conducting and disseminating our research we had the opportunity to discuss the use of the technology with nutritionists, nurses, and physicians from multiple health facilities. One of the first questions that came up in all of these discussions was whether or not 3D imaging could be used to measure individuals with limited mobility, such as persons with a movement disorder or those who are bedridden. We did not identify any study that used 3D imaging to measure people with limited mobility, but the potential is there, and this is another area for future research.

### Increasing measurements

We described how the garment industry was an early supporter of 3D imaging for anthropometry because clothing design requires a large number of measurements. In the health sector, anthropometry is primarily limited to a few measurements: weight and height for everyone, head circumference for newborns, waist circumference for pregnant women, and midupper arm circumference for screening children in low-resource settings. The use of other measurements has been stymied by the practical constraints of manual measurement, and 3D imaging for anthropometry may provide an opportunity to routinely collect a few more measurements with known diagnostic value while developing new indicators with greater predictive power.

Waist circumference provides an illustrative example of improving the collection of measurements with known diagnostic value. Recommendations for measuring waist circumference extend beyond pregnant women. The waist circumference to height ratio is considered a better predictor of morbidity risk than BMI for adults (48), and a WHO expert committee recommended the use of waist circumference alongside BMI for nutritional assessment of nonpregnant individuals (49). Despite a substantial amount of evidence and advocacy for the use of waist circumference, the measurement is not common in regular nutritional screening because it is not easy to measure and is often unreliable. 3D imaging could make measuring waist circumference easier and more reliable, and perhaps more importantly, 3D imaging could capture waist circumference and every other measurement of interest in the same or less time than it takes to collect one measurement with manual equipment, leading to the incorporation of multiple measurements into regular nutritional assessment.

We found 2 examples of researchers developing novel anthropometric indicators on the basis of 3D measurements (40, 46). Although the development of novel indicators based on 3D measurements is an exciting and potentially important application of 3D imaging for anthropometry, the 3D measurement capability is also important for current nutritional indicators. Percentage body fat is an example of a current indicator of nutritional status based on 3D measurements. Body fat can be estimated from weight and body volume, but volume is extremely difficult to accurately estimate with manual, one-dimensional measurements. Currently, researchers use relatively expensive tests (air-displacement plethysmography and DXA) to measure body volume, and percentage body fat is not a part of regular nutritional assessment. For routine assessment, we rely on proxies of body fat based on one-dimensional measurements that often lack predictive power, such as the use of height for BMI. Multiple studies concluded that calculations of percentage body fat on the basis of 3D scan–derived measurements of body volume are both reliable and accurate (36–38). In our validation study, we did not evaluate body fat, but the lower-cost imaging system was able to produce estimates of body volume. An area of future research is to determine if lower-cost imaging systems can make current indicators based on 3D measurement, such as body fat, a part of routine nutritional assessment.

## Conclusions

3D imaging is a standard measurement tool for collecting anthropometry for garment design and ergonomics, but the 3D scanners used in national sizing surveys may not be suitable for regular nutritional assessment in the health sector because of the price and the need for dedicated space. In the health sector, 3D imaging for anthropometry is already used in research and for specialized purposes. The focus of health sector research has been the creation of improved anthropometric indicators based on 3D measurements, but in everyday clinical practice the use of 3D imaging is limited to orthotics. The recent development of lower-cost, portable 3D scanners may have helped to remove barriers to the use of 3D imaging for regular nutritional assessment, and the development of software specifically designed for full-body imaging of children made it possible to measure young children with a 3D scanner. 3D imaging has the potential to reduce the burden on anthropometrists and young children, to improve anthropometric data quality, and to increase the diagnostic value of routine nutritional assessment. However, to our knowledge, there have been no studies on improving anthropometric data quality with 3D imaging. We identified only 4 studies that used lower-cost 3D scanners for anthropometry (including our own), and as would be expected from the general dearth of evidence, many knowledge gaps remain and there are not yet any institutional recommendations for the use of 3D imaging for anthropometry. The Bill and Melinda Gates Foundation and the US CDC are currently building on our validation study by researching the same technology (after recalibration to improve accuracy) in household surveys in low- and middle-income countries. We recommend additional research with the use of lower-cost, single 3D scanners to develop or test novel indicators and to measure waist circumference, body fat, and populations with limited mobility. In addition, to determine if 3D imaging is appropriate for regular nutritional assessment in the health sector, we recommend research in a health facility setting that analyzes barriers, cost, and the ability of 3D imaging to improve quality.

## Notes

Published in a supplement to *Advances in Nutrition*. Presented at the International Union of Nutritional Sciences (IUNS) 21st International Congress of Nutrition (ICN) held in Buenos Aires, Argentina, October 15–20, 2017. The International Union of Nutritional Sciences (IUNS) thanks Mead Johnson Nutrition and Herbalife Nutrition for generously providing grants to support the publication and distribution of the present supplement from the 21st International Union of Nutritional Sciences. The contents of this supplement are solely the responsibility of the authors and do not necessarily represent official views of the IUNS. The supplement coordinators were Angel Gil and Alfredo Martinez. The supplement coordinators had no conflicts of interest to disclose.

Author disclosures: JC and RM, no conflicts of interest. The funding sources had no role in data analyses, data interpretation, or report writing.Publication costs for this supplement were defrayed in part by the payment of page charges. This publication must therefore be hereby marked “advertisement” in accordance with 18 USC section 1734 solely to indicate this fact. The opinions expressed in this publication are those of the authors and are not attributable to the sponsors or the publisher, Editor, or Editorial Board of *Advances in Nutrition*.
